# First hepatitis E outbreak in Southeastern Senegal

**DOI:** 10.1038/s41598-022-22491-8

**Published:** 2022-10-25

**Authors:** Bacary Djilocalisse Sadio, Martin Faye, Marco Kaiser, Maryam Diarra, Fanny Balique, Cheikh Tidiane Diagne, Oumar Faye, Moussa Moïse Diagne, Gamou Fall, Oumar Ndiaye, Cheikh Loucoubar, Abdourahmane Sow, Ousmane Faye, Adama Faye, Cheikh Saad Bouh Boye, Amadou Alpha Sall

**Affiliations:** 1 Virology Department, Institut Pasteur de Dakar, 36, Avenue Pasteur, 220 Dakar, Senegal; 2 GenExpress GmbH, 12103 Berlin, Germany; 3 Epidemiology, Clinical Research and Data Science Unit, Institut Pasteur de Dakar, 220 Dakar, Senegal; 4 OZ Biosciences SAS, 13288 Marseille Cedex 09, France; 5 MIVEGEC (Infectious Diseases and Vector: Ecology, Genetics, Evolution and Control), Univ. Montpelier, IRD, CNRS, 34394 Montpellier, France; 6 West African Health Organisation, 175, Avenue Ouezzin Coulibaly, 01BP: 153, Bobo Dioulasso 01, Burkina Faso; 7 Service de Santé Publique et Appui à la Recherche (CHU Fann), Institut de Santé et Développement, Dakar, Senegal; 8 Université Cheikh Anta Diop de Dakar, Dakar, Senegal; 9 Unité de Recherche et Biotechnologie Microbienne, Faculté de Médecine, de Pharmacie et d’Odontostomatologie (FMPOS), Dakar, Senegal

**Keywords:** Genetics, Risk factors, Infectious-disease diagnostics, Pathogens, Virology, Diseases, Infectious diseases

## Abstract

The Rapid proliferation of traditional gold mining sites in the Kedougou region has led to massive migration of people from neighbouring West African countries and the establishment of several small villages where poor hygiene and sanitation conditions exist. In this context, a Hepatitis E virus outbreak was reported in Kedougou in 2014 with several cases among the traditional mining workers. Herein, we described epidemiological and laboratory data collected during the outbreak’s investigation from February 2012 to November 2014. Any suspected, contact or probable case was investigated, clinical and epidemiological data were collected. In our study, sera were collected and tested for viral RNA and anti-Hepatitis E virus (HEV) IgM. Archived serum samples from Kedougou were retrospectively screened by real-time polymerase chain reaction (RT-PCR) and enzyme-linked immunosorbent assay (ELISA). A total of 65 water samples collected from ponds and wells surrounding gold panners' sites and habitats and 75 tissues samples from rats captured in the environment of traditional gold mining sites were also tested. A total of 1617 sera were collected from 698 suspected cases, 862 contacts and 57 persons with missing information. The median age was 20 (1–88 years-old) and the sex ratio was 1.72. An overall rate of 64.62% (1045/1617) of these patients tested positive for HEV with a high case fatality rate in pregnant women. All water samples and animal tissues tested negative for HEV. Our data help not only determining of the beginning of the HEV outbreak to March 2012, but also identifying risk factors associated to its emergence. However, there is a need to implement routine diagnosis, surveillance and training of health personnel in order to reduce mortality especially among pregnant women. In addition, further studies are needed to identify the virus reservoir and environmental risk factors for HEV in the Kedougou region.

## Introduction

First observed by electron microscopy in 1983 in the stools of patients with non-A and non-B hepatitis, HEV was formally identified in 1990 by cloning its genome from the bile of experimentally infected macaques^1,2^. This led to the complete sequencing of the genome of the Burmese prototype strains^3^. This virus belongs to the family *Hepeviridae* divided into two major genera: *Orthohepevirus* and *Piscihepevirus*^4^ Acute hepatitis E infection mainly includes symptoms such as febrile jaundice, asthenia, anorexia, and nausea-vomiting, and this clinical feature regresses spontaneously within 4–6 weeks^5,6^. However, the infection may become chronic mainly in immunocompromised patients such as those with human immunodeficiency virus (HIV), renal failure or liver transplantation^7^. In rare cases, acute hepatitis E can progress to fulminant hepatitis and lead to death^8^. Although the case fatality rate (CFR) of hepatitis E infection ranges from 0.5 to 4% in the general population^9^, pregnant women are more susceptible to more severe forms of the disease, resulting in a 20% higher CFR in the last trimester of pregnancy^10^. Thus, HEV infection represents the most lethal viral hepatitis during pregnancy^8^.

Fecal–oral transmission is more common and occurs through direct contact or ingestion of contaminated food or water^11^. The epidemiology of HEV therefore differs according to the level of development of a region of the world. Its endemic circulation could be favoured by precarious living conditions including lack of hygiene or health care facilities and limited access to drinking water as is the case in developing countries in Asia and Africa^12^. In developed countries hepatitis E infection was rare and has been reported mainly by travellers returning from trips to HEV-endemic areas such as India, Pakistan, and Bangladesh^13^. However, the number of reported autochthonous cases of HEV infection has increased significantly over the past decade^13^. Thus, HEV infection can present itself in two forms. The first form is the genotypes 1 and 2 infections responsible for the most spectacular epidemics in endemic areas in Asia, Africa and Central America^4^. The second form is sporadic zoonotic infections of genotypes 3 and 4, which occur worldwide but are increasingly reported in industrialized countries^1^.

HEV has been previously responsible for several outbreaks of acute hepatitis in humans worldwide. In 1955, it caused a large waterborne epidemic in New Delhi where 29,300 people were infected with a CFR of 10% in pregnant women^14^. In addition, several outbreaks reported worldwide during the 1990s, and exhibiting the severity of HEV infection in pregnancy^15^, have contributed to considering hepatitis E as a serious public health concern. In Africa, outbreaks of HEV have been previously reported in Sudan and Chad in 2004 with a total of 6861 and 1442 confirmed cases including 87 and 46 deaths, respectively. Overpopulated refugee camps were the most affected sites^16,17^. Epidemics have been also reported in Algeria, Côte d'Ivoire, Ghana, Ethiopia and Somalia^18^.

Since 2009, a surveillance program for acute febrile syndromes was set up by the Institut Pasteur de Dakar (IPD) in the Kedougou region located in Southeastern Senegal^19^. During field investigations around two cases of Rift valley fever, by a collaborating team of the IPD and the medical region of Kedougou, in the Saraya and Kedougou medical districts (Kedougou region) on February 3rd, 2014, several cases of febrile jaundice associated with headache, anorexia and vomiting were identified in the visited mining sites. Most of the suspected cases were living in the villages of Kharakhena, Tenkoto and Bantaco which are the largest gold mining sites in the region. Fulminate forms leading to death as well as maternal deaths were also reported in these villages. Thus, 36 sera were collected and sent to the IPD. A total of 16 out of the 36 sera were confirmed positive for HEV by real-time RT-PCR. Thus, a jointed mission including teams from the Senegalese Ministry of Health and Social actions (MoHSA), the IPD and the Kedougou medical region was organised for rapid identification of suspected cases, investigation around the confirmed cases, contacts tracing and evaluation of risk factors in order to formulate necessary public health recommendations for timely management of patients and outbreak control. Herein, we described epidemiological and laboratory data from the HEV outbreak in the Kedougou region from February to November 2014 and data from retrospective serology and RT-PCR analyses of archive sera collected from February 2012 to January 2014, through the surveillance program for acute febrile syndromes in the aforementioned region.

## Results

### Laboratory testing

A demographic descriptive analysis of the study participants showed that 4.45% were living in Bandafassi, 22.70% in Bantaco, 2.10% in Baytilaye, 0.12% in Dindefelo, 21.89% in Kedougou, 18% in Kharakhena, 0.25% in Ninefesha, 1.30% in Sabodala, 1.18% in Salemata, 3.59% in Saraya, 23.62% in Tenkoto, 0.49% in Thiokoye and 0.31% in Tomboronkoto (Table 1). The median age was 20 (1–88 years-old).

**Table 1:** Demographic descriptive of the study participants.

Mining site	Results of Hepatistis E testing				
	Negative	Positive	Missing	Total	Percentage
Bandafassi	29	43	0	72	4.45
Bantaco	272	95	0	367	22.70
Baytilaye	3	31	0	34	2.10
Dindefelo	1	1	0	2	0.12
Kedougou	106	247	1	354	21.89
Kharakhena	104	185	2	291	18.00
Ninefesha	0	4	0	4	0.25
Sabodala	4	17	0	21	1.30
Salemata	4	15	0	19	1.18
Saraya	9	49	0	58	3.59
Tenkoto	90	292	0	382	23.62
Thiokoye	4	4	0	8	0.49
Tomboronkoto	4	1	0	5	0.31
Total	630	984	3	1617	100

Confirmation of hepatitis E disease was based on positive results of molecular or serological tests. The total number of positive samples using both tests led to a general conclusion named HEV+. The most frequent positive samples were from individuals aged from 16 to 30 years. In our study, 711 out of 1617 sera tested positive for HEV RNA. Indeed to assess the real disease burden, all qPCR-negative sera (n = 906) were tested for anti-HEV IgM serology. A total of 334 sera tested positive for anti-HEV IgM and this makes a total of 1045 HEV positive sera (HEV +). Overall, a rate of 64.62% (1045/1617) of samples tested positive for HEV including 384 contacts, 481 suspected cases and 14 specimens with missing statutory data (Table 2).

**Table 2:** Results from serological and molecular testing.

	IgM				qPCR_ORF2						Hepatitis E (conclusion)		
	N	IgM +	Frequency	p-value (Fisher Test)	Missing	N	ORF2 +	Frequency	p-value (Fisher Test)	Total	HEV +	Frequency	p-value (Fisher Test)
**Age (in year)**													
0–15	303	83	0.229	9.256e−07	6	529	226	0.432	0.241	529	309	0.503	0.132
16–30	381	159	0.49		7	708	327	0.466		708	486	0.568	
31–45	147	65	0.464		3	263	116	0.446		263	181	0.563	
46–89	68	25	0.514		1	107	39	0.368		107	64	0.542	
Missing	7	2	0.667		0	10	3	0.3		10	5	0.5	
Total	906	334				1617	711			1617	1045		
**Sex**													
Female	278	49	0.325	0.078	7	550	272	0.501	0.001	550	321	0.584	0.02
Male	620	117	0.413		10	1054	434	0.416		1054	551	0.523	
Missing	8	–				13				13			
**Status**													
Contact	520	42	0.233	6.02e−10	4	862	342	0.399	5.95e−07	862	384	0.445	3.78e−22
Suspect	336	119	0.536		11	698	362	0.527		698	481	0.689	
Missing	50	7	0.2		2	57	7	0.127		57	14	0.246	
**Outcome**													
Dead	12	5	1	0.002	7	24	12	0.706	0.036	24	17	0.708	0.212
Hospitalized	5	0	0		0	14	9	0.643		14	9	0.643	
Alive	812	143	0.388		9	1456	644	0.445		1456	787	0.541	
Missing	77	19	0.322		1	123	45	0.375		123	64	0.529	
**Pregnant**													
Yes	2	2	1	–	1	11	9	0.9	–	11	11	1	–

The proportion of HEV confirmed either by IgM or qPCR_ORF2 testing was significantly different between age groups (p = 0.132) and independent from sex as much more males than females were included, representing a sex ratio (M/F) of 1.72 (p = 0.02). The number of deaths due to HEV + was 17, representing a mortality of 1.6%. The mortality rate among HEV + pregnant women was 100% (11/11) strongly demonstrating that they are the most vulnerable group within a HEV outbreak (Table 2).

### Spatial and temporal distribution of confirmed hepatitis E cases

**Figure 1:**
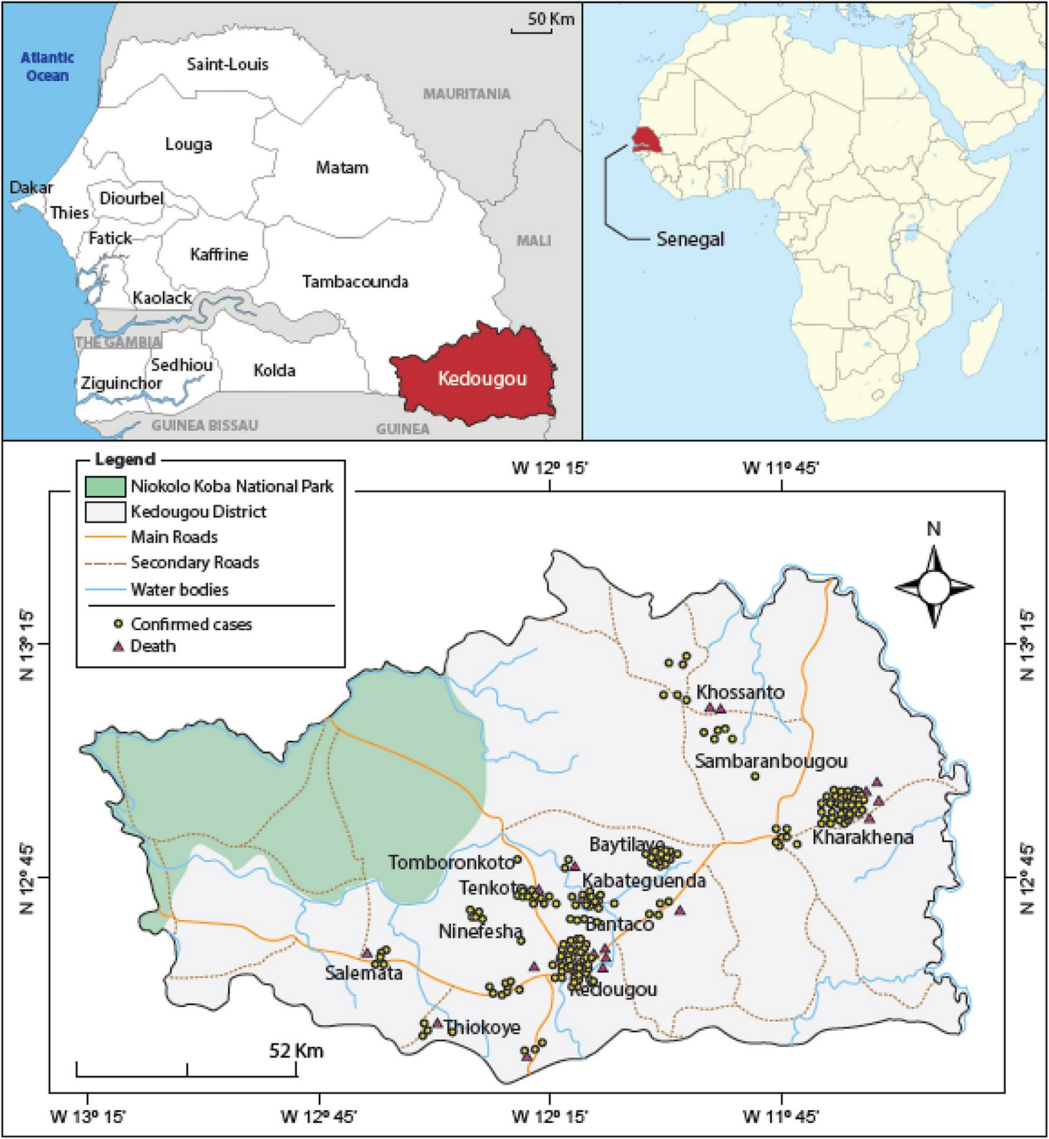
Geographical distribution of confirmed Hepatitis E cases and deaths in the Kedougou region. Confirmed cases are represented by yellow dots while the death are highlighted in red triangles.

The geographical distribution of confirmed hepatitis E cases and deaths in the Kedougou region were also analysed. The majority of confirmed cases were identified from the localities of Kharakhena, Bantaco, Tenkoto and Baytilaye, which are the largest traditional gold mining sites. In addition, some cases were also detected in the locality of Kedougou, where the reference health centre in the region is located. The highest numbers of HEV confirmed deaths were recorded in the localities of Kharakhena and Kedougou (Fig. 1).

**Figure 2:**
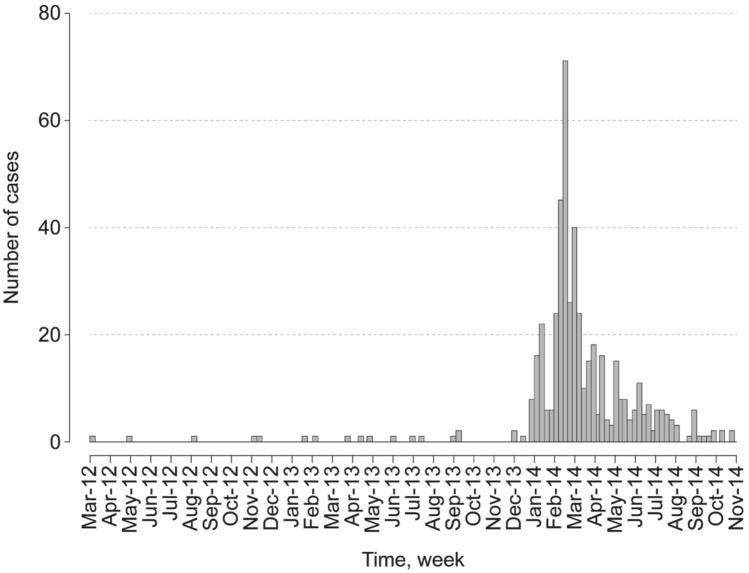
Temporal distribution of confirmed Hepatitis E cases in the Kedougou region from March 2012 to December 2014.

The epidemic curve showed also that the first confirmed cases have been recorded since March 2012 at low noise with a sharp increase in cases in December 2013 and an overall rate of up to 70 cases/100,000 people in February 2014, followed by a sharp decline towards October 2014 (Fig. 2).

### Hepatitis E prevalence in the investigated sites

The HEV prevalence was assessed in each investigated sites in the Kedougou region. The highest rates were recorded in Ninefesha, Sabodala, Salemata, Saraya and Tenkoto villages (p < 1e−05). However, the highest attack rates were observed in the villages of Bantaco (108.68/1000), Tenkoto (121.18/1000) and Kharakhena (27.03/1000), which are the villages with the largest traditional gold mining sites in the Kedougou region. The highest numbers of death were observed in the Kedougou healthcare district (Bantaco, Kedougou and Tenkoto villages), the Saraya district (Kharakhena, Sabodala and Saraya villages) and the Salemata district (Salemata village), with a total of 9, 11 and 1 deaths, respectively. In addition, the disease was 100% lethal in HEV-confirmed pregnant women (Table 3).

**Table 3:** Hepatitis E prevalence through the investigated sites in the Kedougou region.

Mining sites	Population	Cases			p-value	Attack rate (/1000)	Death	Pregnant women	
		N	HEV +	Prevalence				Total	Death
Bandafassi	10,137	72	43	59.72	< 1e-05	7.1	0		
Bantaco	3377	367	64	17.44		108.68	1	1	1
Kedougou	28,160	317	217	68.45		11.26	7	5	5
Kharakhena	10,765	291	135	46.39		27.03	4	1	1
Ninefesha	3441	4	4	100.00		1.16	0		
Sabodala	10,375	21	17	80.95		2.02	4	1	1
Salemata	4496	19	15	78.95		4.23	1	1	1
Saraya	2822	58	45	77.59		20.55	3	2	2
Tenkoto	3152	382	275	71.99		121.19	1		

### Evaluation of associated factors with hepatitis E

Associated factors of HEV infection were also assessed in the Kedougou region, considering parameters such as age, sex or profession. Considering age groups and compared to the under 16 years, the odds for being infected by HEV were more likely among patients 16–30 years-old (AOR = 1.31, 95% CI 1.03–1.65, p-value = 0.025) (Table 4). Regarding the sex, men were 1.32 times more likely to be infected by HEV than female (AOR = 1.32, 95% CI 1.06–1.64, p-value = 0.012). Although lower odds of association between were found between the assessed occupational categories and risk of HEV infection (AOR < 1), assessed significant associations were identified (p-value < 0.05) (Table 4). However, all 65 water samples collected from ponds and wells surrounding gold panners' sites and habitats tested negative for HEV RNA. The 75 tissues organs from rats captured in the environment of traditional gold panning sites during field surveys tested also negative for HEV RNA.

**Table 4:** Evaluation of associated Hepatitis E risk factors with hepatitis E in the Kedougou region.

Characteristics	Adjusted odds ratio (AOR)	CI_95%	Adjusted P-value
**Age group (years)**			
0–15	1	1	–
16–30	1.31	1.03–1.65	0.025
31–45	1.35	0.99–1.84	0.059
> 45	1.26	0.82–1.94	0.288
**Sex**			
Male	1	1	–
Female	1.32	1.06–1.64	0.012
**Profession**			
Others	1		–
Students	0.33	0.20–0.55	< 0.001
Housewives	0.34	0.19–0.59	< 0.001
Gold panners	0.30	0.18–0.50	< 0.001
Labor workers	0.39	0.17–0.89	0.026
Farmers	0.31	0.14–0.71	0.005

### Hepatitis E sequence analysis

**Figure 3:**
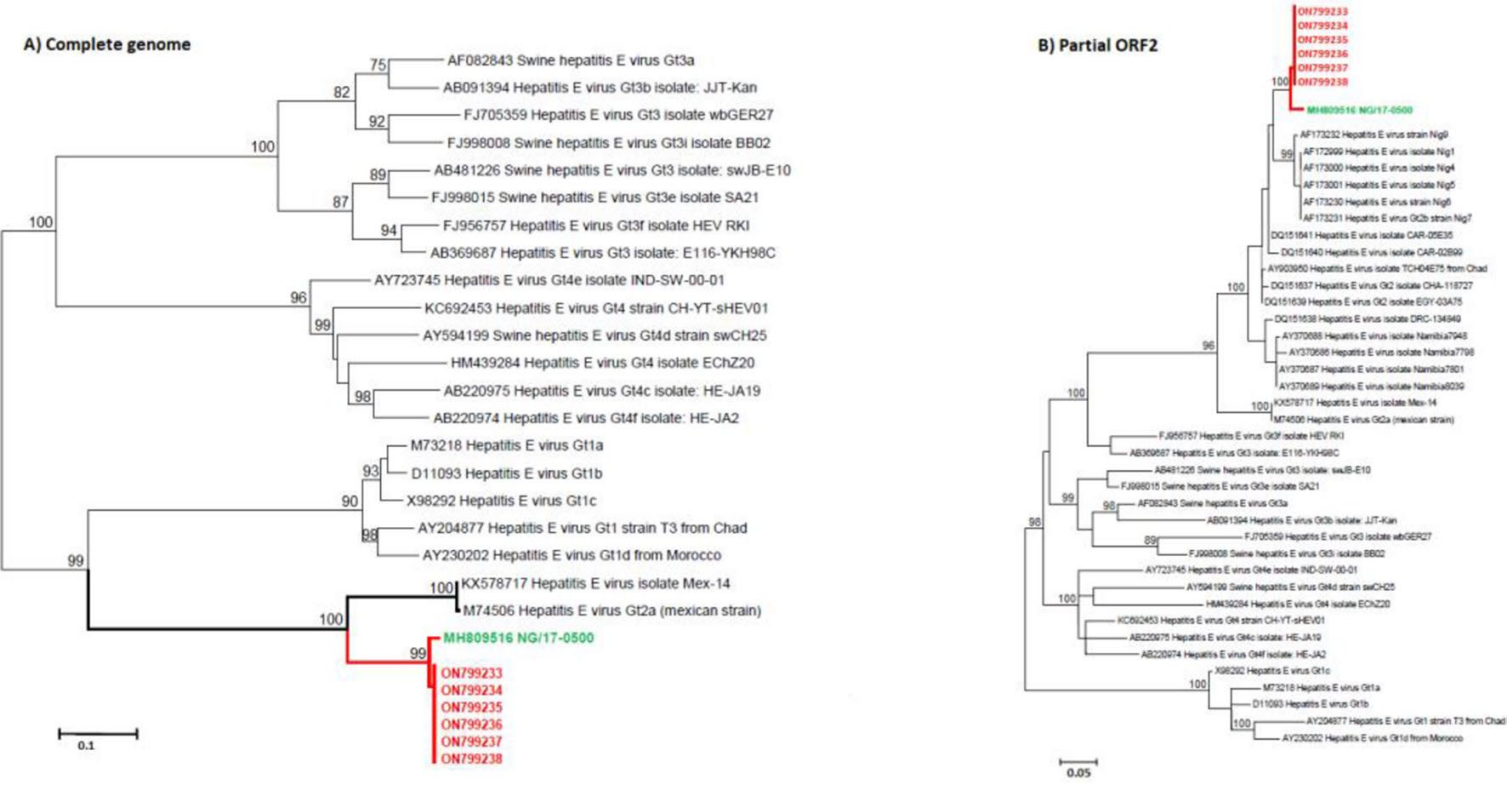
Maximum likelihood (ML) trees of the newly characterized HEV-2b sequences isolated during the 2012–2014 HEV outbreak in Senegal using (**A**) full-length genomes and (**B**) partial ORF2 sequences of HEV. The ML trees were inferred for 1000 replications using the General Time Reversible model with 4 Gamma categories. The ML trees were rooted on midpoints. Nodes were supported by the bootstrap values and values lower than 70% are hidden for more clarity. Strain’s identifiers are designated with accession number, geno/subtype, country and name. The Senegalese sequences are shown in red while the closest sequence from Nigerian (NG/17-0500) is highlighted in green.

For the phylogenetic analysis the near full-length sequence of HEV from 6 samples with high viral load, have been generated by sanger-sequencing. The alignment with 22 complete reference genomes and 38 partial ORF2 sequences for human HEV genotypes demonstrated that all samples are closely related to the 2017 Nigerian strain (MH809516) belonging to the HEV 2b (98% nucleotide identity) (Fig. 3). The newly characterized HEV sequences from Senegal have been deposited in GenBank under the accession numbers ON799233-38.

## Discussion

Hepatitis E virus emerged for the first time in 2014 in Southeastern Senegal, in the Kedougou region. Field investigations allowed confirming the beginning of the HEV outbreak in this region since March 2012, with a significant increase of HEV-confirmed cases from February to March 2014. This HEV emergence could be related to the massive influx of gold diggers in the Kedougou region, particularly in the Kharakhena village where sporadic cases were detected since March 2012 using retrospective molecular and serological assessment in sera from patients with jaundice syndrome.

Cumulative high prevalence of HEV infection in the 16–30 years-old group, men and gold diggers could be associated with their socio-professional activities and are more exposed to poor environmental conditions existing in the traditional mining sites and the mining villages in the Kedougou region. Women have more occupation with the housework. A previous study conducted during a HEV epidemic in Nepal reported also a higher prevalence of the disease in men^20^. Young people were found to be more exposed to the disease in several previous studies^14,20^. However, strong immunity system in adolescents and younger adults included in our study could have contributed to less occurrence of severe hepatic forms of HEV infection as previously reported^20–22^. Other social groups such as students, farmers, workers and housewives might have been exposed to the disease not only as contacts of people returning from the traditional gold mining sites, but also by working temporarily in these sites to gain some money for improvement of their family’s living conditions. The high prevalence of HEV infection identified among contacts (44%) suggested to a possibility of a relatively rapid transmissibility of HEV and pointed to an asymptomatic form of this disease, and the infection shows under diagnosed endemicity during this epidemic in the Kedougou region^23^. This prevalence in apparently healthy people is comparable to previous findings from Nigeria (47.9%)^24^. The high prevalence rate recorded among contacts in this study suggests that HEV infection autochthonously circulated in the Kedougou region, thereby resulting in subclinical infection in the population. Nevertheless, the CFR found during the 2012–2014 HEV outbreak in Kedougou (1.93%) was similar to previously reported data^8^.

Given the novelty in the Kedougou region in 2014 and icteric nature of this disease, the first thought of clinicians is to administer treatment for yellow fever because of the similar clinical hallmark including jaundice. In addition, people infected with yellow fever virus generally visited first the traditional healers and went to healthcare centers only at the terminal phase of the disease. All these factors could explain the high CFR found in our study, especially among pregnant women. Thus, there is a crucial need to establish a differential laboratory diagnosis in the Kedougou region that could help healthcare workers for a more appropriate management of HEV patients. Although the CFR recorded among pregnant women in our study (100%) is higher than previously reported data^25–27^, several studies were unanimous on the severity of HEV infection, especially in pregnant women^25–28^. Therefore, routine diagnosis of HEV in Senegalese pregnant women, especially those living in the Kedougou region, could be implemented during the prenatal visits to reduce the risk of mortality in this vulnerable group.

The increased disease’s incidence observed between December 2013 and January 2014 could be associated with the densification of populations living in the mining villages in very small spaces with really deplorable hygienic conditions for the year-end celebrations. In addition, the highest attack rates found in the localities of Tenkoto, Bantaco, Kharakhena which are the largest traditional gold mining sites where exist poor living conditions (Fig. S1). These densely populated areas are associated with many problems such as high human density confined in a very small space, scarcity of source of potable drinkable water, lack of basic facilities and amenities^24^.

The virus spread to less populated mining villages in the Kedougou and Saraya districts such as Khossanto, Ninefecha, Thiokoye, Tomboronkoto and Cabatenguenda, could be related to a return of indigenous people from the large traditional gold mining sites. Although temporary lock-down of gold mining sites could be the key factor that have driven the shifting of the HEV outbreak epicentre to the Kedougou commune where the last confirmed HEV cases in the region were reported, it had significantly contributed to gradual decrease in the number of confirmed cases. In addition, the awareness on hygiene conditions rolled out by the regional hygiene services and the training of health workers on management of patients have greatly contributed to stop the spread of the HEV disease in Kedougou.

In developing countries, people most often get hepatitis E from drinking water contaminated by feces from people who are infected with the virus^24^. Environmental factors leading the spread of HEV from the mining villages could be related to hygiene conditions and extremely poor sanitation associated with inadequate access to source of drinking water. In this region, gold diggers sometimes use water from unprotected ponds or wells for their own consumption. Social risk factors such as the use of open field (bush) for defecation, the use of pit latrine, presence of latrines in proximity of the most frequent source of drinkable water supply, the total absence of sanitation and hygiene, waste disposal and the housing issues and the attendance to or handling of animals during the end-year celebrations, could have largely contributed to the transmission in the mining villages during the 2012–2014 HEV outbreak in the Kedougou region. Our data also suggested that HEV circulation can evolve from sporadic cases and explode at any given time if hygienic conditions deteriorate. The retrospective serological and molecular testing approach used in our study has not only help determining the beginning of the 2012–2014 HEV outbreak in the Kedougou region, but also provided insights into the real burden of the disease which was highly promoted by the environmental conditions in the gold mining sites. In contrast to a previous study describing the brutal occurrence of HEV epidemics^29^, our data showed that HEV outbreak could be progressive from missed sporadic cases. The Kedougou region holds many gold mining sites where people originating from various West African neighbouring countries such as Mali, Burkina Faso, Guinea Bissau, Guinea Conakry, Nigeria, Niger, Cote d’Ivoire, Ghana, Mauritania and Benin, are living., As the qPCR missed 31.96% of positive samples, dual testing including both serological and molecular methods could be considered for the investigation of future HEV outbreaks. In order to rapidly detect future epidemics, it could be important to implement HEV diagnosis as part of the routine surveillance of febrile syndromes in the Kedougou region and train healthcare workers on the course of this disease to reduce mortality due to HEV in pregnant women. Further studies involving zoology, virology and field epidemiology teams are also needed for identification of the virus reservoir and environmental risk factors for HEV re-emergence in the Kedougou region.

Although foodborne transmission of HEV by the consumption of uncooked/undercooked meat or shellfish has been previously documented^30^, the negative results observed from testing of the source of drinkable water and rodent samples collected during the field investigations, suggested that the source of infection could probably be an imported case from the countries mentioned above.

The novel HEV-2b sequences from the 2012–2014 HEV outbreak in the Kedougou region represents the first complete HEV-2 sequences from Senegal and the second complete HEV-2 sequences Sub-Sahara Africa^31,32^. The genomic data hasn’t allowed identifying the geographical origin of the virus because only few HEV-2 were available from West Africa, so it importantly contribute to our knowledge of the genetic diversity of HEV-2. In addition, these sequences could be useful in future genomic studies focusing on the molecular epidemiology and the natural history of HEV worldwide. They could also serve in the development of therapeutics or antivirals for use in humans.

## Materials and methods

### Ethical statement

The Senegalese National Ethical Committee at the MoHSA approved the surveillance protocol as a less than minimal risk research, and written consent forms were not required. Oral informed consent was obtained from all adults or parents for minors included in this study. Field investigations were regularly supervised by the authorities of the MoHSA in Senegal. Additional samples used in this study were collected in the frame of the national integrated surveillance program of fevers in Senegal and were available from routine diagnostic activities of the World Health Organization (WHO) Collaborating Centre for Arboviruses and Hemorrhagic Fevers at IPD. All methods including the use of human samples, were performed in accordance with the Declaration of Helsinki.

### Study area

The study was conducted in the Kedougou region located in Southeastern Senegal (12°32' N, 12°11' W). Insets are obtained from ArcGIS Desktop V10.8.1 (ESRI 2021, Redlands, CA: Environmental Systems Research Institute; https://desktop.arcgis.com/en/) and sampling sites are placed on base map using their respective XY coordinates and ArcGIS Desktop (Fig. 1). Kedougou is a region with a population of 184,276, 5.1% of whom are under 18 years old, and an average population density of 8 persons per km^2^^33^. The Kedougou region shares its borders with the Republic of Guinea, Mali and The Gambia. The Gambia River and its tributaries flow through the region, some of which dry up during the dry season, creating temporary pools with dense vegetation on the banks. The climate is Sudano-Guinean with a single rainy season from May to October^33^. Temperatures are generally high with an annual average of 28.4 °C. Agriculture is the main economic activity in the region, but hunting and logging are a source of contact between humans and wildlife^33^. Recently, gold mining has become one of the most important economic activities in the region. Field surveys were conducted in the medical districts of Kedougou and Saraya (Fig. 1).

### Case definitions

A suspect case was defined as "any patient living in the Kedougou region between February 2012 and November 2014 with febrile jaundice, anorexia, asthenia, nausea and vomiting associated or not with diarrheal and haemorrhagic manifestations”. Contact case was considered as "any person living in contact with a confirmed or suspected case who may or may not be deceased". A probable case was identified as "any suspect case who died before being tested for markers of HEV infection". A confirmed case was defined as "any suspect case whose specimen tested positive for anti-HEV IgM or viral genome".

### Field activities

During the field investigations, consultation and death records gathered in consultation registers of the health posts located at the military camp of Kedougou, Bantaco, Tenkoto, Syllacounda, Diakhaba, Thiabedji, Bandafassi and the Kedougou and Saraya healthcare centres, were screened for active identification of suspected cases. In addition, investigations were also carried out around confirmed cases including interviews with healthcare staffs and family members for contact tracing. Special attention was also given to the largest gold mining sites such as Kharakhena, Bantaco and Tenkoto where the living conditions are highly suitable for HEV transmission.

### Sample collection

During the investigation, 1582 whole blood samples were collected from February to November 2014. Then 35 additional archived serum samples previously collected from icteric patients as part of the febrile syndrome surveillance program in Kedougou between February 2012 and January 2014, were obtained from the collection of the WHO Collaborating Centre for Arboviruses and Hemorrhagic Fevers at PD. In addition, all patients who consulted during the selected period in one of the health posts of the Kedougou region meeting the inclusion criteria were sampled. The 1617 sera analysed in our study included 698 (44.12%) suspected HEV cases, 862 (54.49%) identified contacts and 57 (3.52%) specimens with missing status data. During the field investigations, a total of 65 water samples were collected from ponds and wells surrounding gold panners' sites and habitats in 1 Litre sterile bottles and 75 rats were captured in the environment of traditional gold mining sites.

### Molecular testing

Whole blood samples were collected and centrifuged in the laboratory to obtain serum. The sera were then aliquoted in small volumes and stored at − 80 °C until further analyses. All 1617 samples were analysed for HEV RNA. Water samples were concentrated using PEG/NaCl method in a final volume of 1 mL and tissues organs from rats euthanized according to the AVMA Guidelines^34^, were triturated in 2 mL of L-15 Medium (Leibovitz). Briefly, RNA was extracted from 140 µL of whole blood, serum samples, concentrated water samples or tissues organs using the QIAamp RNA Viral kit (Qiagen, Heiden, Germany) according to the manufacturer's recommendations. The extracted RNA were tested by real time RT-PCR using a previously described HEV assay targeting the ORF2 gene^35^ with the Quantitect probe kit (Qiagen, Heiden, Germany) according to the manufacturer's instructions. Subsequently, the reaction mix was performed in a final volume of 25 µL containing 5 µL of extracted RNA, 10 µL of buffer (2× QuantiTect Master mix), 1.25 µL of forward and reverse primer, 0.2 of TaqMan probe and 0.2 µL of enzyme mix. Experiments were performed on an ABI 7500 instrument (Applied Biosystem) using the following thermal cycling parameters: 10 min at 50 °C; 15 min at 95 °C, followed by 40 cycles of 15 s at 95 °C and 1 min at 60 °C.

### Sequencing and phylogenetic analysis

Virus RNA from six qRT-PCR positive samples selected according to lowest cycle threshold (Ct) values, was reverse-transcribed using the TaqMan Reverse Transcription Reagents (Thermo Fisher Scientific Life Technologies GmbH, Darmstadt, Germany) with a mixture of random hexamers and cDNA were amplified following the manufacturer’s instructions. Previously published primers^31^ were synthesised by GenExpress and used for the generation of the near full-length sequence. Amplification was performed with Q5 High-Fidelity DNA Polymerase (New England Biolabs, Ipswich, USA) following the manufacturer’s instructions. Amplified libraries were sequenced directly using the BigDye version 3.1 cycle sequencing kit (Thermo Fisher Scientific Life Technologies GmbH), and data were subjected to an automated sequence analysis using a Genetic Analyser 3500 xl Dx (Thermo Fisher Scientific Inc., USA). Sequence and phylogenetic analyses were conducted using the MEGA software version 6.06^36^. Maximum likelihood trees were inferred for 1000 replications using the General Time Reversible model with 4 Gamma categories. Nodes were supported by the bootstrap values and values below 70% are hidden for more clarity.

### Serological testing

All qRT-PCR-negative samples were tested for anti-HEV IgM using the MP Diagnostics HEV IgM ELISA 3.0 (2P35-01 23160-096) kit (Diasorin Murex). A volume of 100 µL of diluted serum (dilution 1:10 in a buffer solution) was added to wells of the polystyrene microplates pre-coated with a highly preserved ORF2’s viral epitope. Strips were then covered and incubated for 1 h at 37 °C for anti-HEV IgM binding to antigens immobilized on the solid support. After incubation, the wells were thoroughly washed to remove unbound elements. A volume of 100 µL of monoclonal antibodies from human anti-Human IgM from mice labelled with horseradish peroxidase were added to each well and strips were incubated for 1 h at 37 °C to allow the formation of antibody-antigen complexes. Strips were again washed for removal of unbound labelled antibodies. A colour-less substrate solution containing TMB (3ʹ–5ʹ-tetrametylbenzidine) was then poured into each well and the plate was incubated at room temperature for 25 min. The presence of specific anti-HEV IgM was indicated by a blue colour. The reaction was then stopped using acid and the reaction’s colour changes to yellow. The intensity of the yellow colour in each well was measured by spectrophotometry at 450 nm using microplate reader (ELx808, BioTek, USA) and is proportional to the amount of anti-HEV IgM present in the sample.

### Statistical analysis

Comparisons for categorical variables were done using the Fisher exact test, with statistical significant set at p < 0.05. Age group are defined as follow: [0–16], [16–30], [30–45] and > 45 years-old. Statistical analysis was performed using univariate binary logistic regression models. Significant variables from the univariate analysis were included to the multivariable binary logistic regression to assess a statistically significant association between the independent variables and the response variable where Adjusted Odds ratio (AOR), 95% confidence intervals (CIs) for AOR, and p-values were used for testing. All statistical analyses and mapping were performed with the R (version 3.2.4) statistical language environment^37^.

## Supplementary Information

Supplementary Information 1.

Supplementary Information 2.

## Data Availability

All data generated or analysed during this study are included in this published article. The newly characterized HEV sequences from Senegal have been deposited in GenBank under the accession numbers ON799233 -38.
