# Supplementary material for: First hepatitis E outbreak in Southeastern Senegal
Source: Sci Rep. 2022 Oct 25;12:17878. doi: 10.1038/s41598-022-22491-8 (PMC9596447; doi:10.1038/s41598-022-22491-8)
Supplement: Supplementary file 1 — Supplementary Information 1. [file 41598_2022_22491_MOESM1_ESM.docx]

**Figure S1**: Poor living conditions existing in the traditional mining sites in the Kedougou region in 2014. **(Kharakhena village)**.
