# Supplementary figures and images for: First hepatitis E outbreak in Southeastern Senegal
Source: Sci Rep. 2022 Oct 25;12:17878. doi: 10.1038/s41598-022-22491-8 (PMC9596447; doi:10.1038/s41598-022-22491-8)

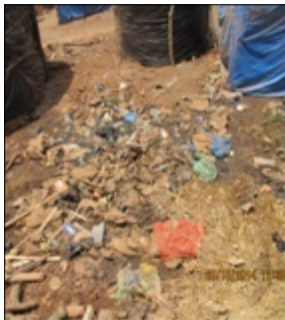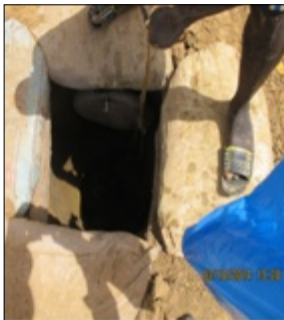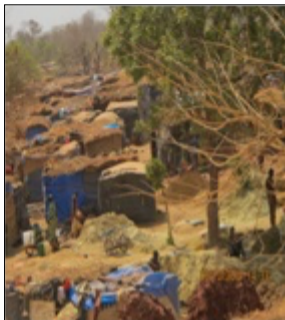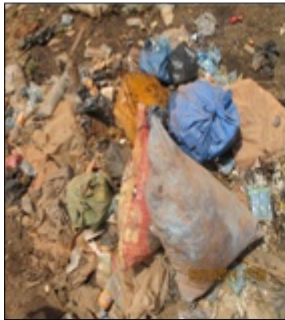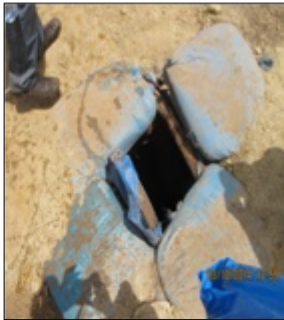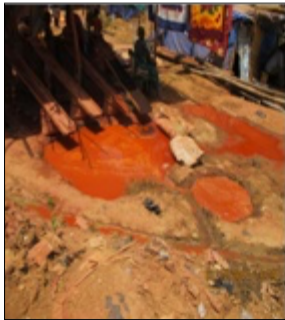

Supplement: Supplementary file 2 — Supplementary Information 2. [file 41598_2022_22491_MOESM2_ESM.pdf]
